# Dietetics and Dyspepsia

**Published:** 1886-08

**Authors:** 


DIETETICS AND DYSPEPSIA. By Sir William Roberts, M. D., F. R. S., Fellow of the Royal College of Physicians. London; Professor of Medicine in the Victoria University; Consulting Physician to the Manchester Royal Infirmary, London: Smith, Elder & Co. New York: G. P. Putnam's Sons. 1886.

This collection of five lectures embraces a kind of knowledge which, as the author says, "is somewhat neglected in these days," but which is becoming more necessary in the practice of medicine. The style of the author is pleasing, and the work abounds in practical points that will make it at once popular with the profession.

